# Does a previous prostate biopsy-related acute bacterial prostatitis affect the results of radical prostatectomy?

**DOI:** 10.1590/S1677-5538.IBJU.2017.0270

**Published:** 2018

**Authors:** Hakan Türk, Sitki Ün, Erkan Arslan, Ferruh Zorlu

**Affiliations:** 1 Department of Urology, Kutahya, Dumlupinar University Evliya Celebi training and Research Hospital, Turkey; 2 Department of Urology, Sivas State of Hospital, Sivas, Turkey; 3 Department of Urology, Harran University Medical School, Sanliurfa, Turkey; 4 Senior Urologist, Izmir, Turkey

**Keywords:** Prostatitis, Prostate, Prostatectomy

## Abstract

**Objective:**

To The standard technique for obtaining a histologic diagnosis of prostatic carcinomas is transrectal ultrasound guided prostate biopsy. Acute prostatitis which might develop after prostate biopsy can cause periprostatic inflammation and fibrosis. In this study, we performed a retrospective review of our database to determine whether ABP history might affect the outcome of RP.

**Materials and Methods:**

441 RP patients who were operated in our clinic from 2002 to 2014 were included in our study group. All patients’ demographic values, PSA levels, biopsy and radical prostatectomy specimen pathology results and their perioperative/ postoperative complications were evaluated.

**Results:**

There were 41 patients in patients with acute prostatitis following biopsy and 397 patients that did not develop acute prostatitis. Mean blood loss, transfusion rate and operation period were found to be significantly higher in ABP patients. Hospitalization period and reoperation rates were similar in both groups. However, post-op complications were significantly higher in ABP group.

**Conclusion:**

Even though it does not affect oncological outcomes, we would like to warn the surgeons for potential complaints during surgery in ABP patients.

## INTRODUCTION

The standard technique for obtaining a histologic diagnosis of prostatic carcinoma (PCa) is transrectal ultrasound (TRUS)-guided prostate biopsy (1). The complication rates related to post-prostate biopsy infections were reported to be 1.7-11.3% (2-5). Biochemical recurrence in radical prostatectomy (RP) patients were deemed as primary treatment failure and recurrence is considered as a sign of PCa. When considering relapse risk, the common variables include preoperative PSA levels, Gleason score, surgical margin status, seminal vesicle and lymph node involvement. While cancer stage affects most of those variables, there are other factors affecting the outcome, such as surgery type and surgeon's experience on the subject including surgical margin status. Acute bacterial prostatitis (ABP) which develops as a complication following TRUS-guided biopsy can cause peri-prostatic inflammation and fibrosis (6, 7). This condition is thought to possibly cause increased perioperative complication rates, difficulty in surgical dissection and positive surgical margins. In this study, we performed a retrospective review of our database to determine whether a history of ABP affected RP outcomes.

## MATERIALS AND METHODS

Data obtained from 1206 patients who were diagnosed with prostate cancer in our clinic were retrospectively reviewed. Out of 482 patients primarily treated with open RP, 11 were excluded from the study due to pre-operative active monitoring. 12 more patients were excluded due to metastasis in removed lymph nodes and were treated with early hormone therapy during post-op period. Out of the rest 459, 18 patients with missing pre-op or post-op data were excluded from the study. Finally, 441 patients who met our criteria and treated with RP in our clinic between 2002 and 2014 were included in our study. All the open radical prostatectomies were done by two different surgeons who were experienced in this type of surgery. Preoperative demographics, operative time, estimated blood loss, transfusion rate, length of stay, margin status, complication rate and reoperation rate were compared between both groups. To categorize complications the recently updated conventional complication classification system of Dindo et al. was used (8). A positive surgical margin was defined as tumor at the inked surface of the specimen. Oncological results were evaluated by staging the operative specimens according to the TNM 2002 classification.

The protocol for infection prophylaxis during prostate biopsy includes two doses of 500mg ciprofloxacin, or if the patient received antibiotic therapy in the last three months, aminoglycosides on the day of biopsy in our clinic. ABP diagnosis was done after the patient showed signs of a fever higher than 38°C, leukocyte presence in urine sediment and proliferation in urine or blood samples (9). The patients presented to our clinic with mainly fever and some other biopsy-related complaints within a mean time period of 36 hours following the prostate biopsy. Patients with suspected ABP were hospitalized in our urology clinic and treated with IV fluids and empirical antibiotics. Later, those patients were treated with the bacteria-specific antibiotics for ABP and discharged.

## RESULTS

There were 41 patients in the prostate biopsy-related acute prostatitis (previous ABP-RP) group and 397 patients in the non-infection (RP) group. Table-1 summarizes the patient demographics and preoperative cancer characteristics. Mean age, pre-op PSA levels and biopsy GS were similar in both groups. A statistically significant difference was detected in the number of patients with clinical stage T1c, T2b and T2c in the RP group in comparison with ABP-RP group. Table-2 lists the intraoperative and perioperative data 86 (21.7%) patients in RP and 18 (40.9%) patients in ABP-RP group required blood transfusion. Mean hospital duration period was 7.12 vs. 7.25 days, mean operation time was 137 vs. 161 minutes in RP and ABP-RP groups, respectively. Mean blood loss, transfusion rate and operation time were found to be significantly higher in ABP-RP group (p=0.041, p<0.001, p<0.001 respectively). Hospital stay duration and reoperation rates were similar in both groups. No post-op mortality was observed in any of the groups. Table-3 lists the pathological features following RP surgery. When post-op complication rates of both groups were compared, ABP-RP group patients had a significantly higher complication rate compared to RP group (Table-4).

**Table 1:** Patient demographics and preoperative cancer characteristics.

		RP	Previous ABP RP	p Value
Mean age (range)		63.2±6.1	63.1±5.5	0.855
Mean PSA (ng/dL) (range)		11.2±8.8	9.6±8.3	0.239
Mean preop Gleason score (range)		6.5±0.7	6.3±0.5	0.655
**No. clinical stage**				
	T1c	328 (82.6)	32 (72.7)	0.02
	T2a	48 (12.1)	10 (22.7)	0.01
	T2b	19 (4.8)	2 (4.5)	0.04
	T2c	2 (0.5)	0	0.01

**Table 2:** Intraoperative and perioperative data.

		RP	Previous ABP RP	p Value
Mean estimated blood loss (mL)(range)		300 (110-950)	410 (150-1250)	0.041
**No. transfusion (%)**				
	Yes	86 (21.7)	18 (40.9)	0.001
Mean days length of stay		7. 2	7.25	0.868
Mean mins operative time (range)		137	161	0.02
No. reop (%)				
	Yes	8 (2)	1 (2.2)	0.771

**Table 3:** Pathological features after radical prostatectomy.

		RP	Previous ABP RP	p Value
Mean prostate vol (cc) (range)		42(31-84)	44(28-75)	0.085
**No. pathological stage (n) (%)**				
	T2a	175 (44)	11(25)	0.01
	T2b	38 (9.5)	15 (34)	0.01
	T2c	82 (20.6)	10 (22.7)	0.061
	T3a	50 (12.5)	8 (18.1)	0.02
	T3b	52 (13)	0	0.001
**No. margin status (n)(%)**				
	Pos	102 (25.7)	8 (18.2)	0.291

**Table 4:** Perioperative and postoperative complications.

Minor (n) (%)		RP	Previous ABP RP	p Value
Retention		12 (3)	4 (9)	0.001
Anastomotic leakage		16 (4)	3 (6.8)	0.023
Simple urinary tract infection		39 (9.8)	7 (15.9)	0.01
Lymphocele		5 (1.2)	1 (2.2)	0.031
Phlebitis		8 (2)	2 (4.5)	0.001
Ileus		7 (1.7)	0	0.001
Superficial abscess		25 (6.2)	5 (11.3)	0.001
**Major (n) (%)**				
	Bowel Injury	3 (0.7)	2 (4.5)	0.001
	Urosepsis	4 (1)	1 (2.2)	0.025
	Cardiac	3 (0.7)	0	0.001
	Acute renal failure	5 (1.2)	1 (2.2)	0.01
	Bladder injury	2 (0.5)	1 (2.2)	0.001
Totals		**131 (32.9)**	**27 (61.3)**	**0.001**

## DISCUSSION

The gold standard in the diagnosis of PCa is TRUS-guided prostate biopsy. However, as with any other diagnostic technique, this technique also has some complication risks (3-5). One of the possible complications is ABP. The effect of prostate biopsy-related ABP on RP is still unclear. To the best of our knowledge, there are no studies that dealt with previous ABP in RP surgery. In theory, a previous infection might cause fibrosis in the prostate and surrounding tissues; which might further cause problems during surgery, stuck tissues and positive surgical margins.

Even though it is not directly related to ABP, there are some studies which reported an increased intraoperative and post-op morbidity rate in patients with previous TURP history prior to RP (7, 10-12). We think that the same is possible in patients with previous ABP history prior to RP. To the best our knowledge, our study is the first series to date which compared RP patients with ABP history with a cancer-matched control group in terms of preoperative, intraoperative, post-operative and pathological parameters.

The only significant difference in preoperative variables between the groups was in terms of clinical stage T1c, T2a, T2b and T2c rates. We don't think that is related to acute prostatitis. What's more, pT2b and pT2c patients were higher in RP group without ABP, which might affect this patient group in terms of surgical margins.

There was a statistically significant difference between the groups in terms of transfusion rate and blood loss, which was expected. In addition, there was a significant difference also in operation times. We think that this was caused by the increased difficulty in dissection, due to obscured planes which were caused by periprostatic inflammation and fibrosis from previous ABP (6, 7). Since there are no previous studies on ABP effect, the closest studies we have are the studies that reviewed the RP patients with TURP. One of those studies reported longer operation times in RP patients with TURP history compared to non-TURP RP patients (13). As a precaution for decreasing the inflammation amount, we systemically wait at least for 2 months between acute ABP and RP in our clinic.

Major and minor early-term complications were seen in higher rates in ABP-RP group compared to the other group. A possible cause for this higher complication rate in ABP-RP group might be the scarring and fibrosis caused by the previous infection, which leads to poor tissue healing at anastomosis. However, it is still not possible to say that all the complications are related to previous acute bacterial prostatitis. Cardiac complications (postoperative myocardial infarction) were seen in only 3 patients, with RP group having the higher number. Higher complication rates were also observed in post-TURP PR series, similar to our study (7, 10-14).

One of the most important parameters that affect biochemical recurrence and local recurrence is positivity of surgical margins (15). In studies which deal with post-TURP RP reported higher rates of surgical margin positivity and related biochemical recurrence (6, 7, 15). One of the possible explanations for this was inability to clearly identify surgical margins due to periprostatic inflammation and difficulty in dissection of surgical planes due to present fibrosis (7).

However, there are other studies which argue that there is no significant difference in terms of surgical margins (11-13). Yet still, all studies come with the presumption that RP surgery is more difficult in patients with previous history of prostate surgery compared to other patients. In our study, we did not detect a significant difference between the groups in terms of surgical margin positivity. Nevertheless, we still think that extra caution should be taken during RP in patients with a history of ABP. Another limitation in our study is the lack of biochemical recurrence and reoccurrence rate's assessment. However, biochemical recurrence is not one of the main objectives researched in this study.

The waiting period between prostate biopsy and RP is still debated. In our clinic, we prefer to wait for 8 weeks following prostate biopsy for controlling the inflammation. This period can be extended in patients with ABP, which can be interpreted as a potential limitation of our study. Although it can be useful to determine an optimal waiting period between ABP and RP, this is not the current study's goal. Other limitation of our study is the small number of ABP patients compared to the other group and the study's retrospective design. Yet another limitation is that the surgeons were not classified according to radical prostatectomy volume. In the literature, experience of the surgeon was shown to affect the surgical margin positivity (15). Nonetheless, we think that our study can be deemed as a compass study for the following studies on this subject.

## CONCLUSIONS

In this retrospective study, we compared perioperative and postoperative complication rates and pathological results of the patients with ABP history prior to RP and normal RP patients. In our results, we detected a significant difference in perioperative hemorrhage, transfusion rates, operation time and post-op complication rates in ABP history prior to RP. No difference was found in terms of surgical margin positivity. Even though it does not seem to affect oncological outcomes, the surgeons must be aware of potential complications during surgery in patients with previous ABP.
